# Proteomic and phosphoproteomic analysis of polyethylene glycol-induced osmotic stress in root tips of common bean (*Phaseolus vulgaris* L.)

**DOI:** 10.1093/jxb/ert328

**Published:** 2013-10-11

**Authors:** Zhong-Bao Yang, Dejene Eticha, Hendrik Führs, Dimitri Heintz, Daniel Ayoub, Alain Van Dorsselaer, Barbara Schlingmann, Idupulapati Madhusudana Rao, Hans-Peter Braun, Walter Johannes Horst

**Affiliations:** ^1^The Key Laboratory of Plant Cell Engineering and Germplasm Innovation, Ministry of Education, College of Life Science, Shandong University, Jinan 250100, PR China; ^2^Institute for Plant Nutrition, Leibniz Universität Hannover, Herrenhäuser Strasse 2, 30419 Hannover, Germany; ^3^Yara GmbH, Hanninghof 35, 48249 Dülmen, Germany; ^4^Applied Research and Advisory Service Agro, K+S KALI GmbH, Bertha-von-Suttner-Strasse 7, 34131 Kassel, Germany; ^5^Institut de Biologie Moléculaire des Plantes (IBMP), 28 rue Goethe, CNRS-UPR2357, Université de Strasbourg, 67083 Strasbourg, France; ^6^Laboratoire de Spectrométrie de Masse Bio-Organique, Université de Strasbourg, IPHC, 25 rue Becquerel, 67087 Strasbourg, France; ^7^Institute of BioPhysics, Leibniz Universität Hannover, Herrenhäuser Strasse 2, D-30419 Hannover, Germany; ^8^International Center for Tropical Agriculture (CIAT), AA 6713, Cali, Colombia; ^9^Institute of Plant Genetics, Leibniz Universität Hannover, Herrenhäuser Strasse 2, D-30419 Hannover, Germany

**Keywords:** Apoplast, cell wall, common bean, dehydrin, phosphoproteomics, proteomics, root tips.

## Abstract

Previous studies have shown that polyethylene glycol (PEG)-induced osmotic stress (OS) reduces cell-wall (CW) porosity and limits aluminium (Al) uptake by root tips of common bean (*Phaseolus vulgaris* L.). A subsequent transcriptomic study suggested that genes related to CW processes are involved in adjustment to OS. In this study, a proteomic and phosphoproteomic approach was applied to identify OS-induced protein regulation to further improve our understanding of how OS affects Al accumulation. Analysis of total soluble proteins in root tips indicated that, in total, 22 proteins were differentially regulated by OS; these proteins were functionally categorized. Seventy-seven per- cent of the total expressed proteins were involved in metabolic pathways, particularly of carbohydrate and amino acid metabolism. An analysis of the apoplastic proteome revealed that OS reduced the level of five proteins and increased that of seven proteins. Investigation of the total soluble phosphoproteome suggested that dehydrin responded to OS with an enhanced phosphorylation state without a change in abundance. A cellular immunolocalization analysis indicated that dehydrin was localized mainly in the CW. This suggests that dehydrin may play a major protective role in the OS-induced physical breakdown of the CW structure and thus maintenance of the reversibility of CW extensibility during recovery from OS. The proteomic and phosphoproteomic analyses provided novel insights into the complex mechanisms of OS-induced reduction of Al accumulation in the root tips of common bean and highlight a key role for modification of CW structure.

## Introduction

Common bean (*Phaseolus vlugaris* L.) is the major food legume for human nutrition worldwide and is a major source of calories and protein, particularly for people in low-income food-deficit countries in the tropics (Graham, 1978; Rao, 2001; Beebe, 2012). Under field conditions, common bean often experiences different abiotic stresses including drought, toxicities of aluminium (Al) and manganese, low soil fertility, and high temperatures (Thung and Rao, 1999; Ishitani *et al.*, 2004; Beebe, 2012). About 60% of common bean-growing areas in the world are affected by drought stress, consequently resulting in a low level of average global yield production (Graham and Ranalli, 1997; Beebe *et al.*, 2008).

In plants growing in dry soil, both shoot and root growth are hampered (Westgate and Boyer, 1985; Sharp *et al.*, 1988). An important feature of the root system response to soil drying is the maintenance of root elongation at low water potentials that can severely inhibit shoot growth (Sharp *et al.*, 2004; Yamaguchi and Sharp, 2010), thus facilitating water uptake from the subsoil (Sponchiado *et al.*, 1989; Serraj and Sinclair, 2002). Investigations of the spatial distribution of the response of root elongation to drought have indicated that elongation is preferentially maintained at the root apex (Sharp *et al.*, 2004; Yamaguchi and Sharp, 2010; Yamaguchi *et al.*, 2010). Physiological studies on the response of the primary root growth of maize to water stress have demonstrated the involvement of three possible mechanisms: osmotic adjustment, modification of cell-wall (CW) extension properties and abscisic acid (ABA) accumulation (Sharp *et al.*, 2004; Yamaguchi and Sharp, 2010). Water loss from the plant cells controls turgor pressure and directly affects the extensibility of the plant CW (Moore *et al.*, 2008). The effect of drought stress on CW structure and properties has been studied extensively in maize roots at physiological and molecular levels (Wu and Cosgrove, 2000; Fan and Neumann, 2004; Sharp *et al.*, 2004; Fan *et al.*, 2006; Poroyko *et al.*, 2007; Zhu *et al.*, 2007; Spollen *et al.*, 2008; Yamaguchi and Sharp, 2010).

Our previous physiological studies in common bean demonstrated that the polyethylene glycol (PEG)-induced osmotic stress (OS) reduction of CW porosity enhanced Al resistance by the reduction of Al accumulation in the root tips (Yang *et al.*, 2010), the main Al-sensitive root zone (Rangel *et al.*, 2007). A transcriptomic analysis indicated that, among the OS-regulated genes, CW synthesis and organization-related genes were mostly downregulated, of which the CW modification genes *XTH* (xyloglucan endotransglucosylase/hydrolase) and *BEG* (glucan endo-1,3-β-glucosidase or β-1,3-glucanase), and the structural protein hydroxyproline-rich glycoprotein (HRGP) were supposed to be involved in modification of CW porosity (Yang *et al*., 2011, 2013). However, transcriptomic profiling sometimes fails to unequivocally reveal regulations of biological processes, for example due to gene redundancy or post-translational modifications (Toorchi *et al.*, 2009; Zörb *et al.*, 2010). Post-translational modifications, such as phosphorylation and glycosylation, can result in a dramatic increase in proteome complexity without a concomitant increase in gene expression (Jensen, 2004; Rose *et al.*, 2004; Jiang *et al.*, 2007). Therefore, in this study, a proteomic and phosphoproteomic approach was applied to: (i) better understand the PEG-induced changes of OS and CW-related proteins in the root tips of Al-sensitive common bean genotype VAX 1, and (ii) further classify the potential mechanisms of CW proteins involved in the adjustment of CW porosity.

## Materials and methods

### Plant materials and growing conditions

Seeds of common bean genotype VAX 1 (Al sensitive) were germinated on filter paper sandwiched between sponges. After 3 d, uniform seedlings were transferred to a continuously aerated simplified nutrient solution containing 5mM CaCl_2_, 1mM KCl, and 8 µM H_3_BO_3_ (Rangel *et al.*, 2007). Plants were cultured in a growth chamber under controlled environmental conditions with a 16/8h light/dark cycle, 27/25 °C day/night temperature, 70% relative air humidity, and a photon flux density of 230 µmol m^–2^ s^–1^ of photosynthetically active radiation at mid-plant height. The pH of the nutrient solution was gradually lowered to 4.5 within 2 d. The plants were then transferred into a simplified nutrient solution (see above) without or with PEG 6000 (Sigma-Aldrich Chemie, Steinheim, Germany). The osmotic potential of the PEG 6000 (150g l^–1^) solution was –0.60MPa, measured with a cryoscopic osmometer (Osmomat 030; Gonotec, Berlin, Germany).

### Measurement of root-elongation rate

Two hours before the treatment was initiated, tap roots were marked 3cm behind the root tip using a fine point permanent marker (Sharpie blue; Stanford), which did not affect root growth during the experimental period. Afterwards, the plants were transferred into a simplified nutrient solution (see above) without or with PEG. Root elongation was measured after the treatment period using a millimetre scale.

### Determination of cell sap osmotic potential

The root tip cell sap was extracted and measured according to Tabuchi *et al.* (2004) with modifications. After treating the plants with PEG (0 and 150g l^–1^) for 24h, 30 root tips of 1cm length were excised and transferred into microfiltration tubes with a membrane pore size of 0.45 µm (GHP Nanosep MF Centrifugal Device; Pall Life Sciences, Ann Arbor, USA) in a 1.5ml plastic tube after removing the free solution on the surface of roots by brief centrifugation. The samples were immediately frozen in liquid nitrogen and stored at –80 °C until use. The root tips were thawed at room temperature, and then centrifuged at 5000*g* for 10min at 4 °C. More than 50 µl of cell sap was obtained from 30 root tips. The osmotic concentration of the cell sap was determined with a cryoscopic osmometer (see above), and the osmotic potential was calculated according to the van’t Hoff equation (Nobel, 1991): π=–*n*R*T*, where π is the osmotic potential, R the gas constant, *T* the absolute temperature, and *n* the molar concentration.

### Extraction of total soluble protein

Approximately 200 root tips of 1cm length were harvested after treating the plants without or with 150g l^–1^ PEG for 24h, and ground with a mortar and pestle in liquid nitrogen. The homogenized sample powder was suspended in 4ml extraction buffer [500mM Tris/HCl, 50mM EDTA, 100mM KCl, 700mM sucrose, 25mM sodium fluoride, 1mM sodium molybdate, 50mM sodium pyrophosphate, 2% (v/v) β-mercaptoethanol and protease inhibitor (1 tablet per 10ml aliquot; Sigma-Aldrich Chemie)] and incubated for 10min on ice. Afterwards, an equal volume of water-saturated phenol was added and incubated for another 10min at room temperature on a rotary shaker. The aqueous and organic phases were separated by centrifugation for 10min at 11 000*g* and 4 °C. The phenolic phase was re-extracted with an equal volume of extraction buffer and centrifuged again. Phenol phases were combined and supplemented with 5 vols of 0.1M ammonium acetate in methanol and incubated overnight at –20 °C for protein precipitation. After centrifugation at 11 000*g* for 3min at 4 °C, precipitated proteins were washed three times with ammonium acetate in methanol and finally with acetone. Pellets were air dried. Extracted proteins were redissolved in rehydration solution (see below) for two-dimensional gel electrophoresis analysis. The protein concentration of extracts were determined in rehydration solution using a 2-D Quant Kit^©^ (GE Healthcare, Munich, Germany) according to the manufacturer’s instructions.

### Extraction of apoplastic proteins

Apoplastic proteins were extracted from control (no PEG) and PEG-treated root tips of 1cm length of bean genotype VAX 1 for 24h, according to the methods described by Zhu *et al.* (2006). Approximately 2000 root tips (1cm length) were excised and transferred into 20mM ice-cold K_2_PO_4_ solution (pH 6.0). The root tips were then rinsed twice with 0.01M MES (pH 5.5) buffer and oriented vertically with the root apex at the top in a filter column with a membrane pore size of 0.45 µM (Macherey-Nagel, Düren, Germany). The filter column was placed into a vial and the whole assembly was held on ice. Twenty millilitres of ice-cold 0.01M MES buffer (pH 5.5) containing 0.2M KCl plus protease inhibitors (1mM phenylmethylsulphonyl fluoride and 5 µl of protease inhibitor cocktail; Sigma) was added to the vial, submerging the plant tissue. The whole assembly containing the root tips was vacuum infiltrated at –50 kPa for 15min and for another 5min without vacuum. The vial was removed, drained, and excess buffer was blotted away from the root tips through the bottom of the filter column. The filter column with root tips was then transferred to a new vial and centrifuged for 15min at 1000*g*. All steps were conducted on ice or in a cold room at 4 °C. Infiltration and centrifugation were then repeated twice. Apoplastic extracts of the resulting three fractions were combined in Vivaspin 6 Centrifugal Concentrators (5000 molecular weight cut-off PES; Vivascience, UK) and centrifuged for 2h at 5000*g*. Precipitation of apoplastic proteins was done as described in the previous section.

In each fraction of apoplastic protein extract, the activity of malate dehydrogenase (MDH), a commonly accepted marker for cytosolic contamination, was assayed according to Bergmeyer and Bernt (1974) and the protein yield was quantified according to Bradford (1976).

### Two-dimensional isoelectric focusing (2D-IEF)/SDS-PAGE

2D-IEF/SDS-PAGE was carried out as described by Führs *et al.* (2008). For IEF, the IPGphor system (GE Healthcare) and immobiline DryStrip gels (18cm) with a non-linear pH gradient of 3–11 (for total soluble and apoplastic proteins) or 4–7 (for total soluble phosphoproteins) were used. Proteins (about 1000 and 80 µg for total and apoplastic soluble proteins, respectively) were dissolved in rehydration solution [8M urea, 2% (w/v) CHAPS, 0.5% (v/v) carrier ampholyte mixture (IPG buffer pH 3–11 or 4–7 NL; GE Healthcare), 50mM dithiothreitol, 12 µl ml^–1^ of DeStreak (GE Healthcare) and a trace of bromophenol blue] and loaded onto individual gel strips. Focusing was done according to Werhahn and Braun (2002). Afterwards, the focused IPG strips were incubated with equilibration solution [50mM Tris/HCl (pH 8.8), 6M urea, 30% (v/v) glycerol, 2% (w/v) SDS, and bromophenol blue] supplemented with either 1% (w/v) dithiothreitol or 2.5% (w/v) iodoacetamide each for 15min. The strips were then placed horizontally onto second-dimension SDS gels and the proteins separated according to Schägger and von Jagow (1987). Afterwards, for total soluble and apoplastic proteins, 2D gels were stained with colloidal Coomassie blue (CBB) G250 according to Neuhoff *et al*. (1985, 1990).

For phosphoprotein detection, 2D gels were stained using the Pro-Q Diamond Phosphoprotein Stain (Pro-Q DPS; Molecular Probes) according to Agrawal and Thelen (2009). Each treatment consisted of three independent biological replicates.

### Image acquisition, image analysis, and statistical analysis

Image acquisition of CBB-stained gels was done using an Epson Expression 1600 scanner (Epson, Mehrbusch, Germany) at 300 dpi. Images were stored as TIFF files. Phosphoprotein detection on gels was done using a Typhoon^TM^ Variable Mode Imager using 532nm excitation and 580nm bandpass emission filters at 100 µm resolution. Images were stored as 16-bit TIFF files (Agrawal and Thelen, 2009). 2D gel image analysis of CBB- and phosphoprotein-stained gels was carried out using ImageMaster^TM^ 2D Platinum Software 6.0 (GE Healthcare). To compensate for variability owing to sample loading, gel staining and destaining, relative spot volumes were calculated (ratio of individual spot volume and total volume of all spots). In this study, significantly changed proteins between treated and control plants were defined as proteins that were more or less abundant by a factor of 1.5 or 0.67, respectively, with *P*≤0.05 (Student’s *t*-test) unless otherwise specified.

### Mass spectrometry (MS) analysis and data interpretation

After manually picking of protein spots with changed abundance, each spot was dried under vacuum. In-gel digestion was performed with an automated protein digestion system (MassPREP Station; Micromass, Manchester, UK). The gel slices were washed three times in a mixture containing 25mM NH_4_HCO_3_:CH_3_CN (1:1, v/v). The cysteine residues were reduced by the addition of 50 µl of 10mM dithiothreitol at 57 °C and alkylated by the addition of 50 µl of 55mM iodacetamide. After dehydration with acetonitrile, the proteins were cleaved in the gel with 40 µl of 12.5ng µl^–1^ modified porcine trypsin (Promega, Madison, WI, USA) in 25mM NH_4_HCO_3_ at room temperature for 14h. The resulting tryptic peptides were extracted with 60% acetonitrile in 0.5% formic acid, followed by a second extraction with 100% (v/v) acetonitrile.

Nano-liquid chromatography (LC)-MS/MS analysis of the resulting peptides was performed using an Agilent 1100 series HPLC-Chip/MS system (Agilent Technologies, Palo Alto, USA) coupled to an HCT Ultra ion trap (Bruker Daltonics, Bremen, Germany). Chromatographic separations were conducted on a chip containing a Zorbax 300SB-C18 column (75 µm inner diameter×150mm) and a Zorbax 300SB-C18 (40 nl) enrichment column (Agilent Technologies).

The HCT Ultra ion trap was calibrated externally with standard compounds. The general MS parameters were as follows: capillary voltage, –1750V; dry gas, 3.0 l min^–1^; and dry temperature, 300 °C. The system was operated with automatic switching between MS and MS/MS modes. The MS scanning was performed in the standard-enhanced resolution mode at a scan rate of 8100 *m*/*z* s^–1^ with an aimed ion charge control of 100 000 in a maximal fill time of 200ms, and a total of four scans were averaged to obtain an MS spectrum. The three most abundant peptides and preferentially doubly charged ions were selected on each MS spectrum for further isolation and fragmentation. The MS/MS scanning was performed in the ultrascan resolution mode at a scan rate of 26 000 *m*/*z* s^–1^ with an aimed ion charge control of 300 000, and a total of six scans were averaged to obtain an MS/MS spectrum. The complete system was fully controlled by ChemStation Rev. B.01.03 (Agilent Technologies) and EsquireControl 6.1 Build 78 (Bruker Daltonics) software. Mass data collected during LC-MS/MS analyses were processed using the software tool DataAnalysis 3.4 Build 169 and converted into.mgf files. The MS/MS data were analysed using MASCOT 2.2.0. (Matrix Science, London, UK) to search against an in-house-generated protein database composed of protein sequences of Viridiplantae downloaded from http://www.ncbi.nlm.nih.gov/sites/entrez (on 6 March 2008) concatenated with reversed copies (decoy) of all sequences (23 478 588 entries). Spectra were searched with a mass tolerance of 0.5Da for MS and MS/MS data, allowing a maximum of one missed cleavage by trypsin and with carbamidomethylation of cysteines, oxidation of methionines, and N-terminal acetylation of proteins specified as variable modifications. Protein identifications were validated when at least two peptides with high-quality MS/MS spectra (Mascot ion score >31) were detected. In the case of one-peptide hits, the score of the unique peptide must be greater (minimal ‘difference score’ of 6) than the 95% significance Mascot threshold (Mascot ion score >51). For the estimation of the false-positive rate in protein identification, a target-decoy database search was performed (Elias and Gygi, 2007).

Protein identifications by MS only were carried out for one of the three gel replicates, because gels obviously were very similar. Also, all analyses allowed us to identify unambiguously proteins of the expected molecular mass range.

### Cellular immunolocalization of dehydrin

After treating the 3-d-old seedlings of common bean genotype VAX 1 without or with PEG 6000 (150g l^–1^) in the simplified nutrient solution (pH 4.5) for 24h, transverse sections (~100–300 µm) of root segment (1–3, 3–6, and 6–9mm from the root apex) were free-hand sectioned with a razor blade and collected in a fixative solution containing 4% paraformaldehyde in 50mM PIPES, 5mM MgSO_4_, and 5mM EGTA (pH 6.9). For the plasmolysis of cells, the free-hand-sectioned transverse root sections were first incubated in 0.8M manitol for 30min on ice before transferring them to the fixative solution. After 1–2h of fixation at room temperature, the samples were washed repeatedly with PBS (pH 7.4) and blocked with 0.2% bovine serum albumin (BSA) in PBS for 30min. The samples were then incubated in a diluted (1:100) solution of primary antibody (rabbit polyclonal anti-dehydrin; Agrisera, Sweden) overnight at 4 °C. The antibody dilution was made with PBS containing 0.2% BSA. The primary antibody was thoroughly washed off the samples with PBS three times for 5min each. Next, they were incubated for 2h in the presence of a 50-fold-diluted solution of the secondary antibody, anti-rabbit IgG coupled with fluorescein isothiocyanate (FITC) (Acris Antibodies, Germany). The samples were washed as described above, mounted on glass slides, and examined under a confocal laser-scanning microscope (Leica TCS SP2; Leica Microsystems, Heidelberg, Germany). Images were captured using Leica Confocal Software.

### One dimensional (1D) SDS-PAGE and fluorescent western blotting

Approximately 50 root tips of 1cm length were harvested after treating the plants without or with 150g l^–1^ PEG for 24h, and ground with a mortar and pestle in liquid nitrogen. The proteins were fractionated in three steps: (i) the homogenized sample powder was suspended in 200 µl of extraction buffer (see above) and incubated for 10min on ice, then centrifuged at 12 000*g* for 15min and the supernatant collected as fraction I; (ii) the remaining residue was resuspended in 200 µl of extraction buffer (see above) and incubated for 10min on ice, then centrifuged at 12 000*g* for 15min to wash the water-soluble proteins away, and the supernatant collected as fraction II; (iii) the remaining residue after step (i)i was resuspended in 200 µl of extraction buffer (see above) containing 1% SDS (to solubilize the hydroponic membrane protein), and incubated for 10min and then centrifuged at 12 000*g* for 15min, and the supernatant collected as fraction III. The proteins were precipitated with ammonium acetate and redissolved in rehydration solution. The protein concentration of extracts was determined using a 2-D Quant Kit^©^ (GE Healthcare) according to the manufacturer’s instructions. Approximately 45, 7, and 4 µg of proteins in fractions I, II, and III was obtained, respectively.

The same volume of the extracted proteins in fractions II and III with fraction I in each corresponding sample (~20, 3, and 2 µg proteins in fractions I, II, and III, respectively) was loaded onto the gel and electrophoretically separated in 12% separating and 4% stacking polyacrylamide gel, and then transferred to a nitrocellulose membrane. After blocking in TBS/Tween 20 containing 1% BSA, the blot was treated with the primary antibody (rabbit polyclonal anti-dehydrin) diluted 1:1000, followed by the secondary antibody (anti-rabbit IgG–FITC) diluted 1:500 separately. Photographs were obtained with a Bio-Imaging System MF-ChemiBIS 4.2 (Benthold, Bad Wildbad, Germany).

To determine the effect of PEG on the abundance of dehydrin protein, CBB-stained Rubisco was used as a loading control in addition to protein quantification. There was no difference between the PEG treatments (data not shown), suggesting that PEG did not affect the abundance of dehydrin.

### Statistical analysis

A completely randomized design was used, with 4–12 replicates in each experiment. If not mentioned otherwise, statistical analysis was carried out using SAS 9.2. Means were compared using a *t*-test or Tukey test depending on the number of treatments being compared. *, **, ***, and ns denote significant differences at *P*<0.05, *P*<0.01, *P*<0.001, and not significant, respectively.

## Results

**Fig. 1.**
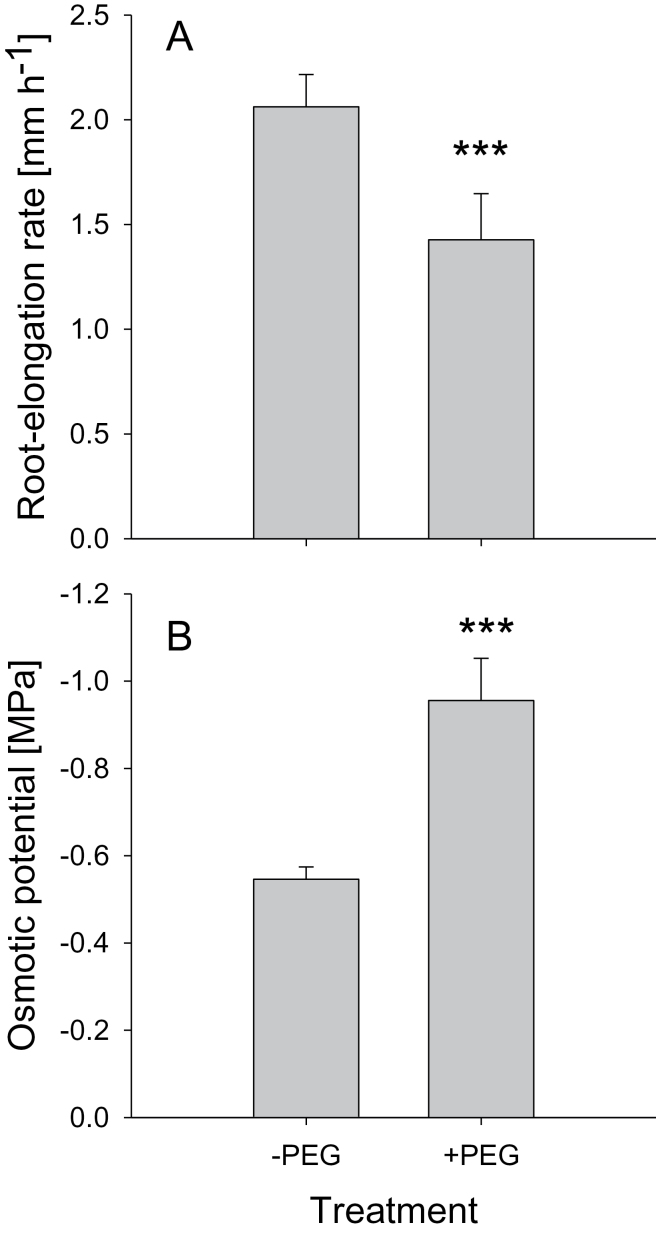
Root-elongation rate (A) and cell sap osmotic potential (B) of 1cm root tips of the common bean genotype VAX 1 under OS (left column, 0; right column –0.60MPa osmotic potential). Plants were pre-cultured in a simplified nutrient solution containing 5mM CaCl_2_, 1mM KCl, and 8 µM H_3_BO_3_ for 48h for acclimation and pH adaptation, and then treated without or with PEG (150g l^–1^) in the simplified nutrient solution (pH 4.5) for 24h. Bars represent means ±SD, *n*=12, for (A) and *n*=4 for (B). *** denotes significant differences between the +PEG treatment and the –PEG control at *P* < 0.001.

Root-elongation rate of bean genotype VAX 1 was inhibited by 31% by a 24h exposure to 150g l^–1^ PEG (pH 4.5) in the simplified nutrient solution (Fig. 1A). Since cell expansion is determined by osmotic potential as well as CW extensibility in the root cells, and osmotic adjustment plays a major role in plant adaptation to OS, the osmotic potential of the root cells was measured using an osmometer. The osmotic potential of the cell sap of the root tips was decreased by 24h treatment with 150g l^–1^ of PEG from –0.55 to –0.96MPa (Fig. 1B), suggesting osmotic adjustment of root cells by accumulating osmolytes facilitating water uptake into cells and thus adaptation to PEG-induced OS.

**Fig. 2.**
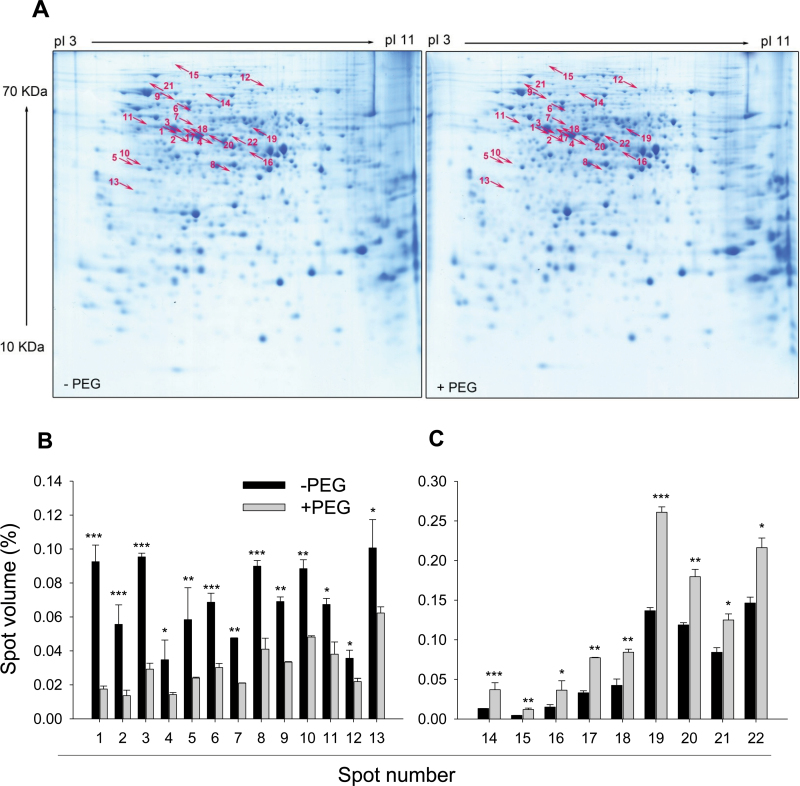
Representative CBB-stained 2D-IEF/SDS-PAGE gels of total soluble proteins (A) and the relative spot volumes of the 22 significantly decreased (B) and increased (C) protein spots in control (–PEG) and PEG-treated root tips of common bean genotype VAX 1. Plants were pre-cultured in a simplified nutrient solution containing 5mM CaCl_2_, 1mM KCl, and 8 µM H_3_BO_3_ for 48h for acclimation and pH adaptation, and then treated without or with PEG (150g l^–1^) in the simplified nutrient solution (pH 4.5) for 24h. Proteins were extracted from the root tips, separated by 2D-IEF/SDS-PAGE, and stained by CBB. Treatment-affected spots were marked by arrows and numbered consecutively. Three independent biological replications of each treatment were analysed using Image Master^TM^ 2D PLATINUM Software 6.0. In (B) and (C), bars represent means ±SD, *n*=3. *, **, and *** denote significant PEG treatment differences at *P*<0.05, *P*<0.01, and *P*<0.001, respectively. (This figure is available in colour at *JXB* online.)

To identify the proteins affected by short-term (24h) PEG-induced OS, the total soluble proteins were extracted from control (–PEG) and PEG-treated root tips. A total of 716 spots were detected after 2D-IEF/SDS-PAGE and CBB staining. Using specific threshold parameters (see Materials and methods), 22 proteins were identified exhibiting differential abundance due to the PEG treatment (Fig. 2A). Nine of these proteins showed higher and 13 proteins showed lower abundance in PEG-treated roots than in control (–PEG) roots (Fig. 2B, C). Close-ups of gel regions containing proteins of differential abundance are shown in Supplementary Fig. S1 (at *JXB* online).

The 22 differentially expressed proteins were analysed by *de novo* peptide sequencing using electrospray ionization (ESI)-MS/MS and identified by sequence comparisons using the National Center for Biotechnology Information (NCBI) protein database (Table 1). The identified 22 proteins were classified according to their proposed biological functions using the UniProt database (Table 1, Supplementary Fig. S2 at *JXB* online). Among the nine increased proteins, the functional categories of carbohydrate metabolism, amino acid metabolism, protein processing, transcription, and unknown were represented by 45, 22, 11, 11, and 11%, respectively. Among the 13 decreased proteins (shown in Table 1), the functional categories of carbohydrate metabolism, amino acid metabolism, other metabolisms, stress response/defence, and protein processing were represented by 38, 31, 15, 8, and 8%, respectively. The functional classification of the identified increased and decreased proteins showed that the majority of the proteins (77%) were involved in pathways of primary metabolism (acetoacetyl-CoA thiolase, fructokinase, *myo*-inositol 1-phosphate synthase, phosphoglycerate mutase, fructokinase-like protein, alcohol dehydrogenase Adh-1, enolase, and NADPH-specific isocitrate dehydrogenase in carbohydrate metabolism; *S*-adenosylmethionine synthetase, d-3-phosphoglycerate dehydrogenase, and methionine synthase in amino acid metabolism), suggesting a pivotal importance of the primary metabolism in the response and adaptation of root tips to PEG-induced OS.

**Table 1.** List of the 22 proteins with significantly different abundance in the root tips of common bean genotype VAX 1 in response to PEGPlants were pre-cultured in a simplified nutrient solution containing 5mM CaCl_2_, 1mM KCl, and 8 µM H_3_BO_3_ for 48h for acclimation and pH adaptation, and then treated without or with PEG (150g l^–1^) in the simplified nutrient solution (pH 4.5). These 22 protein spots are shown in the CBB-stained 2D-IEF/SDS-PAGE gels in Fig. 2 and Supplementary Fig. S1. Spot no. relates to the corresponding spot number of Figs 2 and S1. FC, fold change (the relative protein spot volume of +PEG treatment to the relative protein spot volume of –PEG treatment). The proteins were identified by nano LC-MS/MS and BLASTed in the NCBI database. The protein functions were categorized based on the UniProt database and KEGG pathway. NI, not identified; MW, molecular weight. See Supplementary Table S1 at JXB online for further details.

Spot No.	Identity	MW (Da)	GenBank acc. no.	FC
Carbohydrate metabolism				
4	Acetoacetyl-CoA thiolase (*Medicago sativa*)	41 659.8	ACX47470	0.410
5	Fructokinase (*Arachis hypogaea*)	20 047.7	ACF74294	0.411
7	*myo*-Inositol 1-phosphate synthase (*Phaseolus vulgaris*)	56 431.4	CAH68559	0.441
9	Phosphoglycerate mutase (*Solanum tuberosum*)	60 271.9	AAD24857	0.484
10	Fructokinase-like protein (*Cicer arietinum*)	26 092.1	CAD31714	0.544
16	Alcohol dehydrogenase Adh-1 (*Glycine max*)	34 407.4	AAC62469	2.425
17	Enolase (*Glycine max*)	47 701.9	AAS18240	2.341
18	Enolase (*Glycine max*)	47 701.9	AAS18240	1.986
22	NADPH-specific isocitrate dehydrogenase (*Glycine max*)	49 124.2	AAA33978	1.500
Amino acid metabolism				
1	*S*-Adenosylmethionine synthetase (*Phaseolus lunatu*s)	43 041.9	BAB83761	0.190
2	Methionine adenosyltransferase (*Pisum sativum*)	40 958.3	CAA57581	0.245
3	*S*-Adenosylmethionine synthetase (*Phaseolus lunatus*)	43 041.9	BAB83761	0.307
6	d-3-phosphoglycerate dehydrogenase. putative (*Ricinus communi*s)	63 086.7	EEF43612	0.440
20	*S*-Adenosylmethionine synthetase (*Phaseolus lunatus*)	43 041.9	BAB83761	1.514
21	Methionine synthase (*Glycine max*)	84 266.4	AAQ08403	1.500
Other metabolisms				
12	1-Deoxyxylulose 5-phosphate synthase (*Chrysanthemum x morifolium*)	71 701.7	BAE79547	0.615
13	Inorganic pyrophosphatase, putative (*Ricinus communis*)	33 895.5	EEF44062	0.619
Stress response/defence				
8	Chloroplast thylakoid-bound ascorbate peroxidase (*Vigna unguiculata*)	39 791.4	AAS55852	0.596
Protein processing				
11	Tubulin A (*Glycine max*)	49 552.7	AAX86047	0.566
19	26S proteasome regulatory particle triple-A ATPase subunit1 (*Oryza sativa* Japonica group)	46 667.0	BAB17624	1.910
Transcription				
14	Pre-mRNA-splicing factor SLU7-A *(Arabidopsis lyrata* subsp. Lyrata)	61 961.8	EFH64697	2.794
Unknown				
15	NI	–	–	2.625

To investigate the effect of OS on the regulation of CW proteins of root tips of common bean, soluble and ionically bound apoplastic proteins were extracted and analysed. First, we tested the capability and efficiency of KCl as an extractant of CW proteins from root tips of common bean. Different concentrations were tested for their effect on CW protein yield and cytosolic protein contamination (CPC). The CPC was assessed by measuring MDH activity. CW proteins extracted with 0.1M KCl did not show CPC in each of three sequential extractions; however, the protein yield was low. Infiltration with 0.4M KCl yielded a great amount of protein but also resulted in a high CPC in the first infiltration step (data not shown). Therefore, to obtain a high protein yield with minimum CPC, 0.2M KCl was chosen for extracting CW proteins from PEG-treated root tips of common bean (Table 2). This KCl concentration of 0.2M has been used previously to extract CW proteins from the root-elongation zone of maize (Zhu *et al.*, 2006). Compared with the non-extractable proteins (tightly bound apoplastic proteins and symplastic proteins), MDH activity in the free and CW loosely bound protein fraction was low (Table 2), indicating only low-level contamination. PEG treatment (150g l^–1^) reduced the amount of extractable apoplastic protein in the root tips (Table 2).

**Table 2.** Yield of 0.2mM KCl-extractable apoplastic proteins and the MDH activity (indicator of cytosolic contamination) of extracts from 1cm root tips of common bean genotype VAX 1 grown in absence and presence of PEG 6000 for 24hRoot tips were three times consecutively infiltrated. The residue included tightly bound, non-extractable apoplastic and symplastic proteins.

PEG treatment (g l^–1^)	Infiltration step	Protein yield (ng per 1cm root tip)	MDH activity (nmol min^–1^ per 1cm root tip)
0	I	33.68±5.45	0.10±0.02
	II	29.06±7.66	0.05±0.02
	III	28.86±7.34	0.06±0.03
	Residue	251.19±119.23	0.94±0.54
150	I	21.39±3.01	0.05±0.01
	II	21.51±9.46	0.06±0.03
	III	19.60±7.16	0.04±0.02
	Residue	379.68±212.15	0.58±0.10

**Fig. 3.**
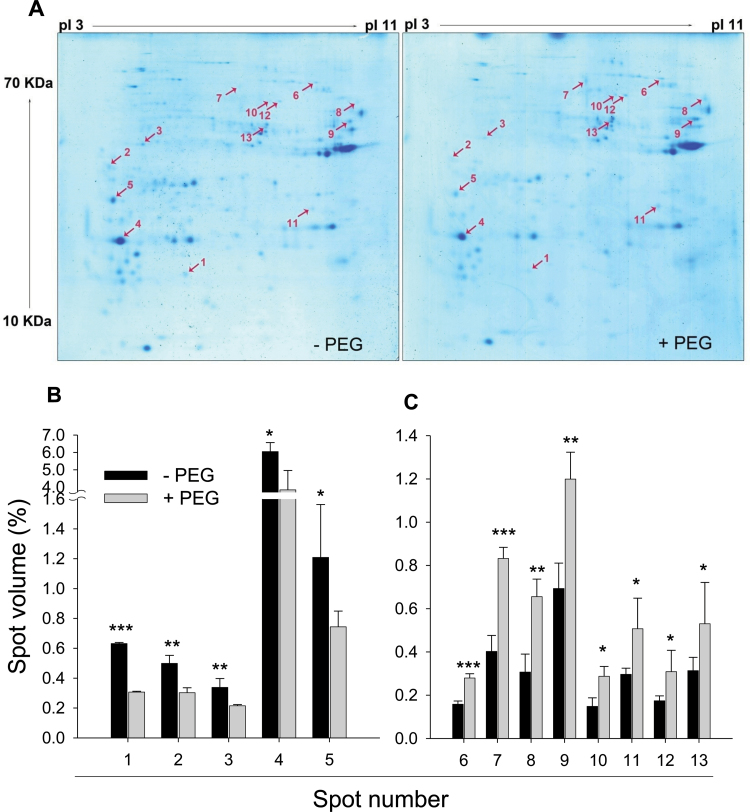
Representative CBB-stained 2D-IEF/SDS-PAGE gel images of apoplastic proteins (A) and the relative volume of the 13 significantly decreased (B) and increased (C) apoplastic protein spots in control (–PEG) and PEG-treated root tips of common bean genotype VAX 1. Plants were pre-cultured in a simplified nutrient solution containing 5mM CaCl_2_, 1mM KCl, and 8 µM H_3_BO_3_ for 48h for acclimation and pH adaptation, and then treated without or with PEG (150g l^–1^) in the simplified nutrient solution (pH 4.5) for 24h. Apoplastic proteins were extracted from root tips, separated by 2D-IEF/SDS-PAGE, and stained by CBB. Treatment-affected spots were marked by arrows and numbered consecutively. Three independent biological replications of each treatment were analysed using Image Master^TM^ 2D PLATINUM Software 6.0. In (B), bars represent means ±SD, *n*=3. *, **, and *** denote significant PEG treatment differences at *P*<0.05, *P*<0.01, and *P*<0.001, respectively. (This figure is available in colour at *JXB* online.)

2D-IEF/SDS-PAGE and subsequent staining with CBB showed that individual proteins could be visualized in spite of the low amount of CW proteins (80 µg) loaded on each gel (Fig. 3). On average, a total of 171 spots were detected on gels containing proteins extracted from PEG-treated and control (–PEG) root tips. A total of 13 proteins were significantly affected by PEG-induced OS. Of these, five and eight proteins showed lower and higher abundance in PEG-treated root tips, respectively (Fig. 3). ESI-MS/MS analysis of these 13 spots allowed the identification of eight proteins by BLAST search at the NCBI protein database (Table 3). Two of the proteins (fructokinase and pathogenesis-related protein 1) were reduced and six proteins (β-xylosidase, pectinacetylesterase precursor, serine hydroxymethyltransferase, serine carboxypeptidase, and fructose-1,6-bisphosphate aldolase) increased in abundance by PEG-induced OS (Table 3). Close-ups of the gels are shown in Supplementary Fig. S3 at *JXB* online.

**Table 3.** List of 13 apoplastic proteins with significantly different abundance in the root tips of common bean genotype VAX 1 in response to PEGPlants were pre-cultured in a simplified nutrient solution containing 5mM CaCl_2_, 1mM KCl, and 8 µM H_3_BO_3_ for 48h for acclimation and pH adaptation, and then treated without or with PEG (150g l^–1^) in the simplified nutrient solution (pH 4.5). These 13 protein spots are shown in the CBB-stained 2D-IEF/SDS–PAGE gels of Figs 3 and Supplementary Fig. S3. Spot No. relate to the corresponding spot number in Fig. 3 and in supplemental Fig. S3. FC: fold change (the relative protein spot volume of +PEG treatment to the relative protein spot volume of -PEG treatment). The proteins were identified by nano LC-MS/MS and BLASTed in NCBI database. NI, not identified; MW, molecular weight. See Supplementary Table S1 at *JXB* online for further details.

Spot no.	Identity	MW (Da)	GenBank acc. no.	FC
1	NI	–	–	0.486
2	NI	–	–	0.607
3	Fructokinase (*Arachis hypogaea*)	20 047.7	ACF74294	0.636
4	Pathogenesis-related protein 1 (PvPR1) (*Phaseolus vulgaris*)	16 511.4	CAA43637	0.635
5	NI	–	–	0.616
6	Beta xylosidase (*Fragaria*×*ananassa*)	83 468.2	AAS17751	1.768
7	NI	–	–	2.067
8	NI	–	–	2.136
9	Pectinacetylesterase precursor (*Vigna radiata* var. Radiata)	43 804.9	CAA67728	1.730
10	Serine hydroxymethyltransferase, putative (*Ricinus communis*)	51 889.9	XP_002522806	1.935
11	Serine carboxypeptidase, putative (*Ricinus communis*)	50 034.7	XP_002521402	1.709
12	Serine hydroxymethyltransferase (*Gossypium hirsutum*)	51 889.9	ACJ11726	1.771
13	Fructose-1.6-bisphosphate aldolase (*Pisum sativum*)	38 473.4	CAA61947	1.692

**Fig. 4.**
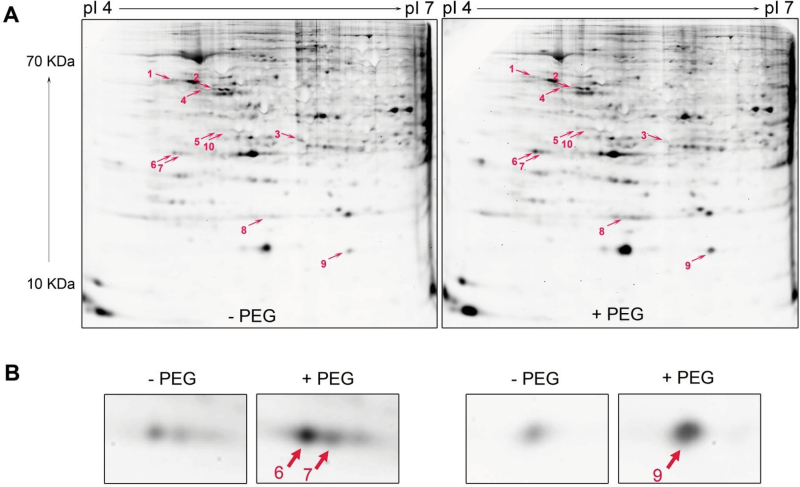
Representative Pro-Q DPS-stained 2D-IEF/SDS-PAGE gels of the total soluble proteins in the control (–PEG) and PEG-treated root tips of common bean genotype VAX 1 (A) and three magnified spots with PEG treatment-enhanced protein abundance (B). Plants were pre-cultured in a simplified nutrient solution containing 5mM CaCl_2_, 1mM KCl, and 8 µM H_3_BO_3_ for 48h for acclimation and pH adaptation, and then treated without or with PEG (150g l^–1^) in the simplified nutrient solution (pH 4.5) for 24h. Proteins were extracted from the root tips, separated by 2D-IEF/SDS-PAGE, and stained by Pro-Q DPS. Treatment-affected spots were marked by arrows and numbered consecutively. Three independent biological replications of each treatment were analysed using the Image Master^TM^ 2D PLATINUM Software 6.0. (This figure is available in colour at *JXB* online.)

The PEG-induced changes of phosphorylated and dephosphorylated proteins in root tips were examined by a phosphoproteomic approach using 2D-IEF/SDS-PAGE and Pro-Q DPS staining. Out of the identified 10 significantly changed proteins, seven showed increased phosphorylation (glucose-6-phosphate isomerase, actin, dehydrin, and lactoylglutathione lyase) and three proteins decreased phosphorylation (Ser/Thr-specific protein phosphatase 2A, regulatory subunit β isoform, pyruvate kinase, and branched-chain amino acid aminotransferase) in response to PEG-induced OS (Fig. 4A, Table 4). Close-ups of three of these proteins with high abundance are shown in Fig. 4B and were identified as belonging to the same protein family, dehydrin. The close-ups of all differentially formed proteins in response to PEG treatment are presented in Supplementary Fig. S4 at *JXB* online.

**Table 4.** List of 10 phosphoproteins with significantly different abundance in the root tips of common bean genotype VAX 1 in response to PEGPlants were pre-cultured in a simplified nutrient solution containing 5mM CaCl_2_, 1mM KCl, and 8 µM H_3_BO_3_ for 48h for acclimation and pH adaptation, then treated without or with PEG (150g l^–1^) in the simplified nutrient solution (pH 4.5). These 10 phosphoprotein spots are shown in the Pro-Q DPS-stained 2D-IEF/SDS–PAGE gels of Fig. 4 and in Supplementary Fig. S4. Spot no. relates to the corresponding spot number of Fig. 4 and Supplementary Fig. S4. FC, fold change (the relative protein spot volume of +PEG treatment to the relative protein spot volume of –PEG treatment). The proteins were identified by nano LC-MS/MS and BLASTed in NCBI database. See Supplementary Table S1 at JXB online for further details. MW, molecular weight.

Spot no.	Identity	MW (Da)	GenBank acc. no.	FC
1	Ser/Thr-specific protein phosphatase 2A A regulatory subunit β isoform (*Medicago sativa* subsp.×varia)	65161.6	AAG29594	0.647
2	Pyruvate kinase (*Lactuca sativa*)	56174.9	ABS87384	0.481
3	Branched-chain amino acid aminotransferase, putative (*Ricinus communis*)	45159.9	XP_002530599	0.541
4	Glucose-6-phosphate isomerase (*Zea mays*)	68403.5	NP_001147983	1.837
5	Actin (*Phaseolus acutifolius*)	33121.3	AAZ95077	2.277
6	Dehydrin (*Phaseolus vulgaris*)	22955.6	AAB00554	2.048
7	Dehydrin (*Phaseolus vulgaris*)	22955.6	AAB00554	1.846
8	Lactoylglutathione lyase, putative/glyoxalase I, putative (*Arabidopsis thaliana*)	20830.4	NP_001030996	2.414
9	Dehydrin (*Phaseolus vulgaris*)	22955.6	AAB00554	2.438
10	Dehydrin (*Phaseolus vulgaris*)	22955.6	AAB00554	1.625

Gene analysis by quantitative real time-PCR (Livak and Schmittgen, 2001) indicated that PEG did not affect the transcriptional regulation of the gene *DHN* (GeneBank accession no. U54703), encoding the dehydrin protein, in the root tips of common bean (data not shown).

**Fig. 5.**
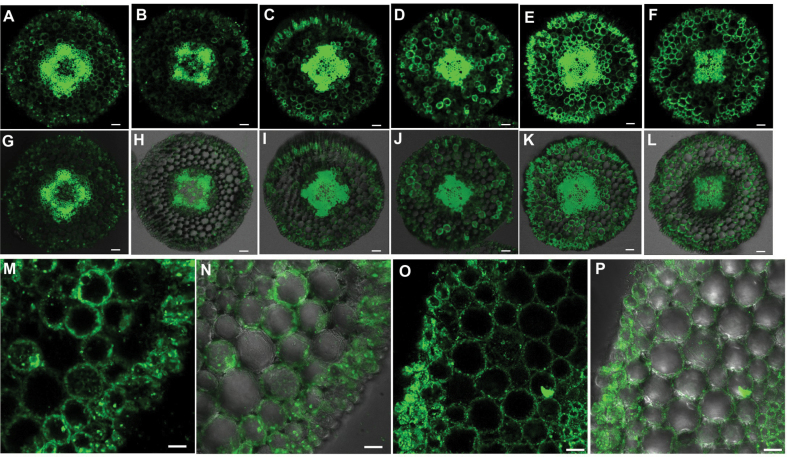
Cellular immunolocalization of dehydrin in the root tips of common bean. Plants were pre-cultured in a simplified nutrient solution containing 5mM CaCl_2_, 1mM KCl, and 8 µM H_3_BO_3_ for 48h for acclimation and pH adaptation, and then treated without or with PEG (150g l^–1^ PEG 6000) in the simplified nutrient solution (pH 4.5) for 24h. Transverse root sections were treated with the anti-dehydrin polyclonal antibody and secondary anti-rabbit IgG–FITC. The images were captured with a confocal laser-scanning microscope (see Materials and methods). (A–F, M, O) Fluorescence of dehydrin; (G–L, N, P) merged image of dehydrin.(A, G, D, J) root apical 0–3mm segments; (B, H, E, K) root apical 3–6mm segments; (C, I, F, L) root apical 6–10mm segments; (M, N) close-up of immunostaining of dehydrin in root apical 3–6mm segments; (O, P) close-up of immunostaining of dehydrin in root apical 3–6mm segments after plasmolysing the cells with 0.8M manitol. (A–C, G–I) No PEG treatment (–PEG); (D–F, J–P) PEG treatment (+PEG). Bars, 50 µm (A–L); 20 µM (M–P).

In a next step, the dehydrin protein was localized in the root apical 0–3, 3–6, and 6–10mm segments using immunofluorescence (Fig. 5). In the absence of PEG, dehydrin primarily localized in the central cylinder, while the signal intensity in the epidermis and cortex was relatively weak, particularly in the 3–6mm region (Fig. 5A–C, G–I). However, when roots were subjected to PEG stress, the signal intensity, particularly in the 3–6mm region, was highly enhanced in the epidermis and cortex of all three root apical regions (Fig. 5D–F, J–L). High resolution of the cross-sections in PEG-treated 3–6mm root apical region shows that dehydrin was localized in the CW or plasma membrane (Fig. 5M, N). To confirm whether dehydrin localized in the CW or plasma membrane, a high concentration of manitol (0.8M) was used to induce plasmo- lysis of cells. The unchanged localization of dehydrin before and after plasmolysis (Fig. 5O, P) suggested that dehydrin is most probably localized in the CW.

**Fig. 6.**
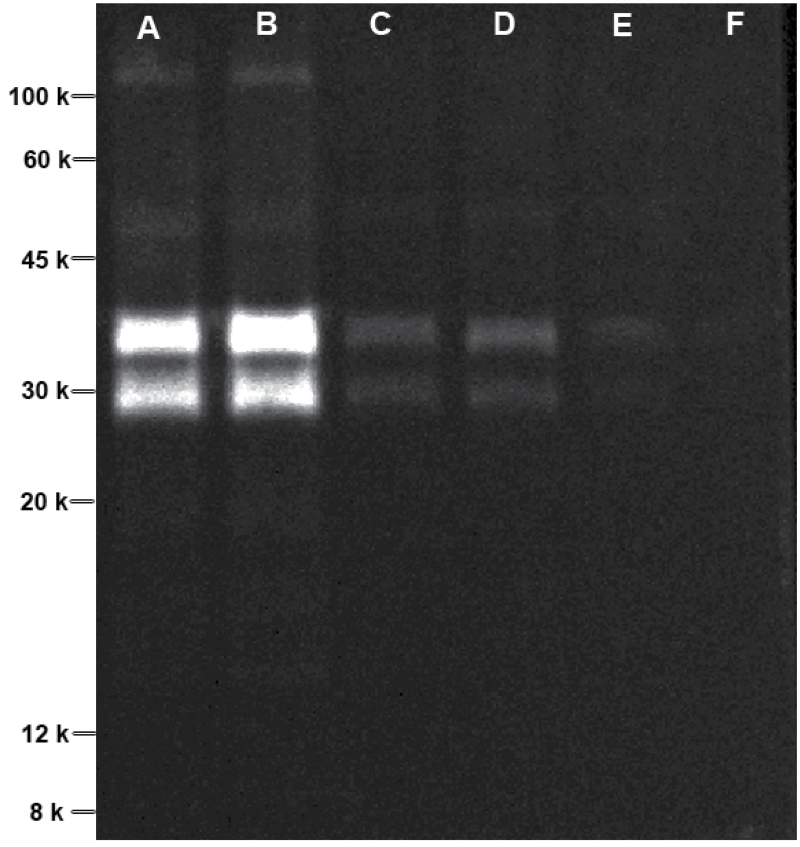
Fluorescent western blotting of dehydrin protein in the root tips of common bean. Plants were pre-cultured in a simplified nutrient solution containing 5mM CaCl_2_, 1mM KCl, and 8 µM H_3_BO_3_ for 48h for acclimation and pH adaptation, and then treated without or with PEG (150g l^–1^ PEG 6000) in the simplified nutrient solution (pH 4.5) for 24h. The proteins from root tips treated without and with PEG were fractionally extracted with protein extraction buffer without and with 1% (w/v) SDS (see Materials and methods). Lanes: A, fraction I of –PEG; B, fraction I of +PEG; C, fraction II of –PEG; D, fraction II of +PEG; E, fraction III (+1% SDS) of –PEG; F, fraction III (+1% SDS) of +PEG.

To verify the primary localization of dehydrin in the CW, we fractionally extracted the root apical protein with and without SDS in the extraction buffer to be able to differentiate between hydrophilic and hydrophobic dehydrin. We found that dehydrin appeared predominantly in the water-soluble protein fraction (Fig. 6, lane A), whereas hardly any dehydrin was found in the water-insoluble protein fraction (Fig. 6, lane E). In addition, PEG treatment did not affect the protein abundance of dehydrin in root tips of bean (Fig. 6, lanes A and B), in agreement with the results of the 2D-IEF/SDS-PAGE of total and apoplastic protein separation (Figs 2 and 3). The two protein bands suggested the presence of two dehydrin isoforms.

Prediction of protein phosphorylation sites by using KinasePhos 2.0 software (http://kinasephos2.mbc.nctu.edu.tw/index.html; Wong *et al.*, 2007) revealed that dehydrin (GenBank accession no. AAB00554) contains 18 serine (S), seven threonine (T), and three tyrosine (Y) predicted potential phosphorylated sites (Supplementary Fig. S5 at *JXB* online).

## Discussion

Physiological osmotic adjustment and modification of CW extensibility have been suggested as two major mechanisms involved in the maintenance of root elongation during water deficit (Sharp *et al.*, 2004; Yamaguchi and Sharp, 2010). In this study, functional categorization of total soluble proteins showed that the majority of the affected proteins were involved in primary metabolism (Supplementary Fig. S2, Table 1), particularly carbohydrate metabolism, confirming the important role of accumulation of carbohydrates and soluble amino acids involved in osmotic adjustment (Morgan, 1984). On the other hand, protein synthesis and modification of carbohydrates regulated by the proteins involved in metabolic pathways may facilitate the adjustment of CW synthesis and extensibility, and thus the regulation of root elongation. Also, some proteins are supposed to play key roles in the protection against OS-induced severe physical destruction of CW integrity, such as dehydrin (Layton *et al.*, 2010), which was highly significantly changed in phosphorylation state by OS and localized in the CW in this study. Therefore, based on the above information, the discussion below will particularly focus on carbohydrate and amino acid metabolism and on CW-related proteins.

The osmotic adjustment in the growing root region in response to OS can in part result from increased net accumulation rates of osmotic solutes. Physiological factors influencing such accumulation rates are solute synthesis, uptake, catabolism, and utilization. All of these represent adaptive responses contributing to growth maintenance (Sharp *et al.*, 2004). It has been reported that OS increases sugar (e.g. fructose, glucose, and sucrose) accumulation in the roots of mung bean (*Vigna mungo*) seedlings (Itoh *et al.*, 1987) and the tropical tree *Colophospermum mopane* (Johnson *et al.*, 1996). Fructokinase specifically catalyses the transfer of a phosphate group from ATP to fructose, thereby activating this sugar for further metabolic processes. In this study, OS-induced reduced abundance of fructokinase in the root tips of common bean (Fig. 2, Supplementary Fig. S1, Table 1) may lead to fructose accumulation, and in this sense may be regarded as part of osmotic adjustment. Indeed, in soybean roots, Toorchi *et al.* (2009) also found by proteomic analysis that PEG-induced OS reduced the formation of a fructokinase 2 protein.

In *Arabidopsis*, Hummel *et al.* (2010) found that organic acids in addition to K^+^ are main contributors to osmotic adjustment under water-deficit conditions. We previously reported that PEG-induced OS increased citrate, malate, *cis*-aconitate, and fumarate contents in root tips of common bean (Yang *et al.*, 2010), indicating a major role for the accumulation of these organic acids in osmotic adjustment. Isocitrate dehydrogenase is an enzyme that participates in the citric acid cycle. It catalyses oxidative decarboxylation of isocitrate to α-ketoglutarate and requires either NAD^+^ or NADP^+^ as co-substrate, producing NADH and NADPH, respectively. It was thought (Chen and Gadal, 1990) that citrate in the cytosol is first converted to isocitrate by the action of aconitase, and then to 2-oxoglutarate by the action of NADP-specific isocitrate dehydrogenase (NADP-ICDH). Subsequently, 2-oxoglutarate is utilized as carbon skeleton for the glutamine synthase/glutamate synthase pathway. Thus, a decrease in NADP-ICDH activity was associated with the accumulation of citrate in the plant tissue (Sadka *et al.*, 2000; Kihara *et al.*, 2003). In contrast, in the present study, it was found that OS increased the expression of NADPH-specific ICDH in root tips of common bean (Fig. 2, Supplementary Fig. S1, Table 1), thus not supporting the hypothesis that increased accumulation of citrate in the cytosol is a consequence of reduced NADP-ICDH activity (Massonneau *et al.*, 2001; Anoop *et al.*, 2003; Rangel *et al.*, 2010). However, on the other hand, enhanced formation of NADPH-specific ICDH due to OS may increase the accumulation of citrate in root tips through increasing citric acid cycle turnover. Moreover, it may be involved in defence processes, since NADP-ICDH was shown to play an important role in cellular defence against stress-induced oxidative injury (Jo *et al.*, 2002; Lee *et al.*, 2002). Liu *et al.* (2010) found that an isolated cDNA encoding cytosolic NADP-dependent ICDH from maize conferred salt tolerance to *Arabidopsis*.

Phosphoglycerate mutase (PGM) is a glycolytic enzyme, which catalyses the conversion of 3-phosphoglycerate to 2-phosphoglycerate. Ergen *et al.* (2009) found that the expression of the gene encoding PGM of wild durum wheat (*Triticum turgidum*) was promoted in leaves while it was suppressed in roots during dehydration. Mazarei *et al.* (2003) observed that an *AtPGM* gene in *Arabidopsis* was localized in the shoot and root meristems, and that expression was downregulated by ABA. However, the function of this protein in stress responses remains to be elucidated. *Myo*-inositol 1-phosphate synthase (MIPS) catalyses the rate-limiting step in the synthesis of *myo*-inositol. It was reported that *mips1* mutants resulted in lowered *myo*-inositol levels and enhanced cell death in *Arabidopsis* (Meng *et al.*, 2009; Donahue *et al.*, 2010). Moreover, Donahue *et al.* (2010) provided evidence that *mips1* mutants had increased sensitivity to ABA and OS (salt and sorbitol treatment), which could be rescued by *myo*-inositol supplementation. The OS-induced suppression of MIPS in this study (Fig. 2, Supplementary Fig. S1, Table 1) suggests that the root tips of common bean suffered severe stress causing cell damage through lowered *myo*-inositol abundance. Enolase is responsible for the conversion of 2-phosphoglycerate to phosphoenolpyruvate, which is involved in glycolysis. Enolase was detected in the CWs of *Candida albicans*, *Arabidopsis thaliana*, *Medicago sativa*, and *Zea mays* (Chivasa *et al.*, 2002; Pitarch *et al.*, 2002; Watson *et al.*, 2004; Zhu *et al.*, 2006). Using immunolocalization, enolase was shown to be secreted to the CW or the extracellular space, even though it lacked a signal peptide (Edwards *et al.*, 1999). In this study, the abundance of enolase was enhanced by PEG treatment (Fig. 2, Supplementary Fig. S1, Table 1). The role of upregulation of enolase under OS remains unclear.

Some proteins involved in amino acid biosynthesis were differentially affected in abundance by OS in this study, such as *S*-adenosylmethionine synthase (methionine adenosyltransferase, SAMS), methionine synthase (MetS) and d-3-phosphoglycerate dehydrogenase (PHGDH) (Fig. 2, Supplementary Fig. S1, Table 1). MetS catalyses the final step in methionine biosynthesis and SAMS catalyses the conversion of ATP and methionine into *S*-adenosylmethionine (SAM) (Ravanel *et al.*, 1998). SAM serves as a co-factor in a variety of biochemical reactions in all living organisms. It acts as a methyl donor to proteins, lipids, polysaccharides, and nucleic acids (Tabor and Tabor 1984), participates in CW lignin synthesis (Sederoff and Chang 1991), and mediates the biosynthesis of ethylene (Yang and Hoffman, 1984; Kende 1993). Also, SAM is believed to play a regulatory role in the synthesis of methionine and other aspartate-derived amino acids (Peleman *et al.*, 1989). Manavella *et al.* (2006) demonstrated that overexpression of the sunflower (*Helianthus annuus*) HD-Zip protein subfamily 1 member *Hahb-4* transcription factor in *A. thaliana* improved desiccation tolerance via the repression of SAMS transcription and 1-aminocyclopropane-1-carboxylate oxidase, and subsequently suppressed the biosynthesis of ethylene. Ingram and Bartels (1996) reported that drought-caused alterations in the chemical composition and physical properties of the CW (e.g. CW extensibility) may involve genes encoding SAMS. Under non-stressed conditions, higher expression of *SAMS* genes correlated with the extent of lignification of tissues in *Arabidopsis* (Peleman *et al.*, 1989). Lignification of CW by methylation of lignin monomers was described as one mechanism to avoid water loss under dehydration (Bhushan *et al.*, 2007). Indeed, it has been reported that water deficit intensified the lignifications of root tip CWs in maize (Fan *et al.*, 2006) and soybean (Yamaguchi *et al.*, 2010). Moreover, it has been suggested that cellular levels of SAM are regulated by MetS activity forming methionine, a precursor of SAM (Ravanel *et al.*, 1998). Therefore, the induction of MetS transcripts suggests an increased production of methionine and lignin methylation by SAM. Recent results confirmed that SAMS was involved in tolerance to abiotic stresses such as salinity (Sanchez-Aguayo *et al.*, 2004; Jiang *et al.*, 2007) and drought stress (Toorchi *et al.*, 2009; Pandey *et al.*, 2010). In this study, PEG treatment led to increased abundance of a MetS (spot 21 in Fig. 2, Supplementary Fig. S1, Table 1) and SAMS (spot 20 in Fig. 2, Supplementary Fig. S1, Table 1), while the amount of a further three SAMS proteins (spots 1, 2, and 3 in Fig. 2, Supplementary Fig. S1, Table 1) in the root tips were repressed. The PEG-induced differential regulation of SAMS proteins may be indicative of different isoforms of this protein. Under OS conditions, the increase in SAMS may either improve lignification of root tip CWs or increase biosynthesis of ethylene, while downregulation of SAMS may be caused by a changed demand for more methyl groups for lignin methylation (Bhushan *et al.*, 2007). It is known that transcriptional and translational levels of SAMS are downregulated by OS (Seki *et al.*, 2002; Yan *et al.*, 2005; Jiang *et al.*, 2007; Toorchi *et al.*, 2009).

Sharp *et al.* (2004) suggested that, in maize, the extent of osmotic adjustment in the primary root tip, although substantial, was insufficient in roots growing under severe drought to maintain turgor comparable to well-watered levels. This was assessed directly by measuring the spatial distribution of turgor using a cell-pressure probe, which showed that turgor was reduced by >50% throughout the root-elongation zone of roots growing at a water potential of –1.6MPa compared with well-watered control plants (Spollen and Sharp, 1991). Thus, enhancement of CW extensibility may contribute to the maintenance of root elongation in the apical region of water-stressed roots (Sharp *et al.*, 2004; Yamaguchi and Sharp, 2010). Under multiple abiotic stress conditions, we recently found that OS-induced reduction of CW porosity could improve Al resistance by restricting Al accumulation in the apoplast of common bean root tips (Yang *et al.*, 2010). Also, by means of a transcriptome analysis, we identified some genes encoding proteins involved in CW modification (xyloglucan endotransglucosylase/hydrolase, XTH) and CW structure (HRGPs), which were supposed to play major roles in the PEG-mediated decrease of CW porosity (Yang *et al.*, 2011). Several CW proteins/enzymes are believed to play roles in modifying the CW structure and controlling CW extension. These include expansin, XTH, and glucanases (Wu and Cosgrove, 2000; Bray, 2004; Sharp *et al.*, 2004; Moore *et al.*, 2008). Therefore, we aimed at identifying these proteins and clarifying their response to PEG treatment by extracting apoplastic and loosely ionically bound proteins in root tips of common bean and separating them by protein electrophoresis techniques. Unlike what we expected, only a low protein yield was obtained by a fractional infiltration method. In addition, the extracted apoplastic protein yield in each infiltration step was reduced in PEG-treated roots (Table 2), further supporting the suggestion that PEG-induced OS reduces CW porosity (Yang *et al.*, 2010), thereby reducing exchangeability of these proteins by extractants. Thus, proteins with a high molecular weight could not be exchanged so that predominantly small molecular proteins were identified in this study. Although Zhu *et al.* (2007) identified a large number of water deficit-induced CW proteins in maize using the same extraction procedures employed in the current study, the type I CW of common bean is different from the type II CW of maize: the former contains higher pectic polysaccharides (Carpita and Gibeaut, 1993), which is the main factor determining CW porosity (Baron-Epel *et al.*, 1988).

In spite of this, 13 PEG-induced differentially affected proteins in the CW were obtained and eight were identified by MS analysis (Fig. 3, Table 3). TOS-induced reduction of fructokinase was also found in the CW fraction. It has been suggested that fructose formed from sucrose cleavage could be directly and rapidly converted into UDP-glucose via the hexose-phosphate pool (i.e. fructose-6-phosphate↔glucose-6-phosphate↔glucose-1- phosphate), and subsequently transferred to the apoplast for synthesis of CW polysaccharides (Konishi *et al.*, 2004). Thus, it appears that a decrease in fructokinase may result in a reduction in CW polysaccharide content in root tips, impeding root growth under OS, since fructokinase catalyses the transfer of fructose into fuctose-6-phosphate. Toorchi *et al.* (2009) also reached a similar conclusion. Indeed, Odanaka *et al.* (2002) reported that suppression of the gene *Frk2* encoding fructose kinase inhibited root growth of tomato. In conclusion, fructokinase may play a role in the regulation of the energy metabolism: (i) by providing fructose-6-phosphate for glycolysis; and/or (ii) through conversion to UDP-glucose to support biosynthesis of CW material (Karni and Aarni, 2002).

Fructose-1,6-bisphosphate aldolase reversibly catalyses the conversion of fructose-1,6-bisphosphate to glyceraldehyde-3-phosphate. The extracellular matrix proteome analysis revealed that fructose-1,6-bisphosphate aldolase was indeed a CW protein, which was increased during dehydration stress in chickpea (*Cicer arietinum*) (Bhushan *et al.*, 2007) and rice (*Oryza sativa*) (Pandey *et al.*, 2010), corroborating the results of this study showing that PEG-induced apoplastic dehydration increased the abundance of apoplastically localized fructose-1,6-bisphosphate aldolase in the root tips of common bean (Fig. 3, Table 3). Enhanced synthesis of fructose-1,6-bisphosphate aldolase was also reported as a response to salt stress in rice (Abbasi and Komatsu 2004) and the mangrove plant *Bruguiera gymnorhiza* (Tada and Kashimura, 2009). Enhanced formation of fructose-1,6-bisphosphate aldolase may increase the flow of carbon through the Calvin cycle and lead to C-skeleton production for subsequent increased carbon flux through glycolysis. These traits would also lead to osmolyte production and contribute to OS tolerance.

Pathogenesis-related proteins (PRs) are mainly involved in the defence against pathogenic constraints and in general adaptation to stressful environments. The CW is the major accumulation site of these PRs (Edreva, 2005). To our knowledge, the biochemical function of PR1 is not fully known. However, suppression of PR1 by OS (Fig. 3, Table 3) in this study may reflect a lower protection level and severe root-cell damage. β-Xylosidase was found in the stock of pathogen-degrading enzymes (Tezuka *et al.*, 1993). A gene, *AtBLX1*, encoding β-xylosidase in *Arabidopsis* was proposed to be involved in secondary CW hemicellulose metabolism and plant development (Goujon *et al.*, 2003). In contrast to our results showing PEG-mediated increased abundance of β-xylosidase in the CW of bean root tips (Fig. 3, Table 3), Zhu *et al.* (2007) found that water deficit reduced β-xylosidase in the apical 3–7mm region of the maize primary roots. The exact function of β-xylosidase in response to osmotic/drought stress remains to be elucidated. In addition, the amount of pectin acetylesterase, serine hydroxymethyltransferase, and serine carboxypeptidase was enhanced by OS, but the role of these in OS is not yet known.

Phosphoproteomics revealed that three proteins showed reduced and seven proteins increased phosphorylation by OS. Of these, dehydrin underwent significantly enhanced phosphorylation by OS (Fig. 4, Table 4), while no PEG effect on abundance of dehydrin by CBB staining (data not shown) and western blotting (Fig. 6) was found, indicating that the activation of dehydrin during OS by phosphorylation may play an important role in root responses to OS. Dehydrins are the second biggest group of late embryogenesis abundant proteins, which are well known to play crucial roles in cellular dehydration tolerance (Ingram and Bartels, 1996; Hundertmark and Hincha, 2008) and desiccation tolerance of angiosperm plants (Gaff and Oliver, 2013). A number of studies have demonstrated that dehydrin proteins play an important role in drought tolerance by preventing membrane denaturation and maintaining the integrity of the CW (Lopez *et al.*, 2003; Collett *et al.*, 2004; Vicré *et al.*, 2004; Samarah *et al.*, 2006; Hu *et al.*, 2010). Dehydrins are known to undergo phosphorylation both *in vivo* and *in vitro* (Heyen *et al.*, 2002; Jiang and Wang, 2004; Alsheikh *et al.*, 2005; Brini *et al.*, 2006; Röhrig *et al.*, 2006).

**Fig. 7.**
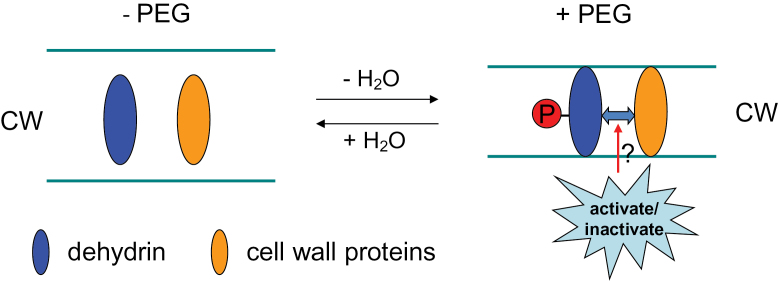
Schematic model of the potential interaction between phosphorylated dehydrin protein and other CW proteins under OS. (This figure is available in colour at *JXB* online.)

Dehydrins were found to be localized in various subcellular sites including the plasma membrane, cytoplasm, and nucleus (Danyluk *et al.*, 1998; Wisniewski *et al.*, 1999; Carjuzaa *et al.*, 2008). Recently, Layton *et al.* (2010) observed that dehydrin accumulated mainly near the CW of dried tissues in the desiccation-tolerant fern *Polypodium polypodioides*. The author supposed that the ability to avoid CW damage in some desiccation-tolerant species may be partially attributed to CW localization of dehydrins enabling reversible CW deformation. The cellular localization of dehydrin in CW was further verified by the present study (Fig. 5, Fig. 6). Dehydrins are extremely hydrophilic proteins (Close *et al.*, 1989), which can attract, sequester, and localize water, and may behave as a lubricant between either the plant CW and cell membrane or between individual CW layers. It has been reported that dehydrins are highly specialized proteins that lack a fixed three-dimensional structure and have evolved to maintain their disordered character under conditions such as water deficit, in which unfolded states of several globular proteins would tend to collapse (Mouillon *et al.*, 2008). However, the effect of phosphorylation on dehydrin structure is small and does not significantly enhance the response to OS induced by glycerol and PEG 4000 *in vitro* (Mouillon *et al.*, 2008). In spite of this, the studies *in vitro* may not reflect the response of dehydrin structure to phosphorylation *in vivo*, since dehydrins may interact with other proteins *in vivo*. In addition, the site of OS induced by glycerol and PEG 4000 is different from PEG 6000 because of the differences in molecular weight; the former solutes mainly induce OS in the cytoplasm, while the latter act additionally in the apoplast (Yang *et al.*, 2010). Thus, it appears that the increased dehydrin formation could play an important role in the protection of CW against breakage and in the maintenance of the mechanical CW integrity. Our previous study (Yang *et al.*, 2010) suggested that CW porosity was reduced by PEG-induced OS and quickly recovered after the removal of OS in root tips of common bean, which was concluded on the basis of the penetration of ions with a different hydrated ionic radius (Al^3+^>La^3+^>Sr^2+^>Rb^+^) into the apoplast, and CW modification proteins XTH, BEG, and the structural protein HRGP may participate in this reversible regulation (Yang *et al.*, 2011). Therefore, the phosphorylated dehydrins may prevent the CW from PEG-caused mechanical disruption by interacting with other CW proteins or may act independently, and consequently maintain the elastic extension (reversible stretching) properties of the CW and thus allow quick reversion of CW extension after removal of OS, as depicted in Fig. 7.

In conclusion, our large-scale proteomic analysis revealed the importance of carbohydrate and amino acid metabolism in the response of root tips to OS. Phosphoproteomics provided novel insights into the potential role of dehydrin as a CW structure modulator, which may participate in alteration of CW porosity by maintaining the integrity and reversible extension properties of the CW during OS.

## Supplementary data

Supplementary data are available at *JXB* online.

Supplementary Fig. S1. Close-ups of significantly decreased and increased total soluble protein spots in response to PEG in the root tips of common bean genotype VAX 1.

Supplementary Fig. S2. Functional categories of the 22 significantly decreased and increased proteins in response to PEG-induced OS in the root tips of common bean genotype VAX 1.

Supplementary Fig. S3. Close-ups of significantly decreased and increased apoplastic protein spots in response to PEG in the root tips of common bean genotype VAX 1.

Supplementary Fig. S4. Close-ups and the relative volume of significantly decreased and increased phosphorylated protein spots in response to PEG in the root tips of common bean genotype VAX 1.

Supplementary Fig. S5. Prediction of potential phosphorylation sites of the dehydrin protein.

Supplementary Table S1. Detailed information for identified proteins including 22 total soluble proteins, 13 apoplastic proteins and 10 phosphorylated proteins, which are listed in Tables 1, 3 and 4, respectively, by nano LC-MS/MS.

Supplementary Data

## Abbreviations:

2D-IEF: two-dimensional isoelectric focusing
ABA: abscisic acid
Al: aluminium
BSA: bovine serum albumin
CPC: cytosolic protein contamination
CW: cell wall
ESI: electrospray ionization
FITC: fluorescein isothiocyanate
LC: liquid chromatography
MS: mass spectrometry
NCBI: National Center for Biotechnology Information
OS: osmotic stress
PEG: polyethylene glycol.
