# Supplementary material for: Proteomic and phosphoproteomic analysis of polyethylene glycol-induced osmotic stress in root tips of common bean (Phaseolus vulgaris L.)
Source: J Exp Bot. 2013 Oct 11;64(18):5569–86. doi: 10.1093/jxb/ert328 (PMC3871817; doi:10.1093/jxb/ert328)
Supplement: Supplementary Data [file supp_64_18_5569__index.html]

Proteomic and phosphoproteomic analysis of polyethylene glycol-induced osmotic stress in root tips of common bean (Phaseolus vulgaris L.) — Proteomic and phosphoproteomic analysis of polyethylene glycol-induced osmotic stress in root tips of common bean (Phaseolus vulgaris L.) — Supplementary Data 

# Proteomic and phosphoproteomic analysis of polyethylene glycol-induced osmotic stress in root tips of common bean (*Phaseolus vulgaris* L.)

## Supplementary Data

Data files

**Files in this Data Supplement:**

- Supplementary Data - Supplementary Data
- Supplementary Data - Supplementary Data
