# Supplementary material for: Proteomic and phosphoproteomic analysis of polyethylene glycol-induced osmotic stress in root tips of common bean (Phaseolus vulgaris L.)
Source: J Exp Bot. 2013 Oct 11;64(18):5569–86. doi: 10.1093/jxb/ert328 (PMC3871817; doi:10.1093/jxb/ert328)
Supplement: Supplementary Data [file supp_ert328_jexbot102137_file001.pdf]

Proteomic and phosphoproteomic analysis of polyethylene glycol-induced osmotic stress in root tips of common bean (*Phaseolus vulgaris* L.)

Zhong-Bao Yang[1,2], Dejene Eticha[3], Hendrik Führs[4], Dimitri Heintz[5], Daniel Ayoub[6], Alain Van Dorsselaer[6], Barbara Schlingmann[7], Idupulapati Madhusudana Rao[8], Hans-Peter Braun[9], Walter Johannes Horst[2*]

[1]The Key Laboratory of Plant Cell Engineering and Germplasm Innovation, Ministry of Education; College of Life Science, Shandong University, Jinan 250100, PR China; [2]Institute for Plant Nutrition, Leibniz Universität Hannover, Herrenhäuser Str. 2, 30419 Hannover, Germany; [3]Yara GmbH, Hanninghof 35, 48249 Dülmen; [4]Applied Research and Advisory Service Agro, K+S KALI GmbH, Bertha-von-Suttner-Strasse 7, 34131 Kassel, Germany; [5]Institut de Biologie Mooléculaire des Plantes (IBMP), 28 rue Goethe, CNRS-UPR2357, Université de Strasbourg, 67083 Strasbourg, France; [6]Laboratoire de Spectrométrie de Masse Bio-Organique, Université de Strasbourg, IPHC, 25 rue Becquerel, 67087 Strasbourg [7]Institute of BioPhysics, Leibniz Universität Hannover, Herrenhäuser Str. 2, D-30419 Hannover, Germany; [8]International Center for Tropical Agriculture (CIAT), AA 6713, Cali, Colombia; [9]Institute of Plant Genetics, Leibniz Universität Hannover, Herrenhäuser Str. 2, D-30419 Hannover, Germany

Supplementary material

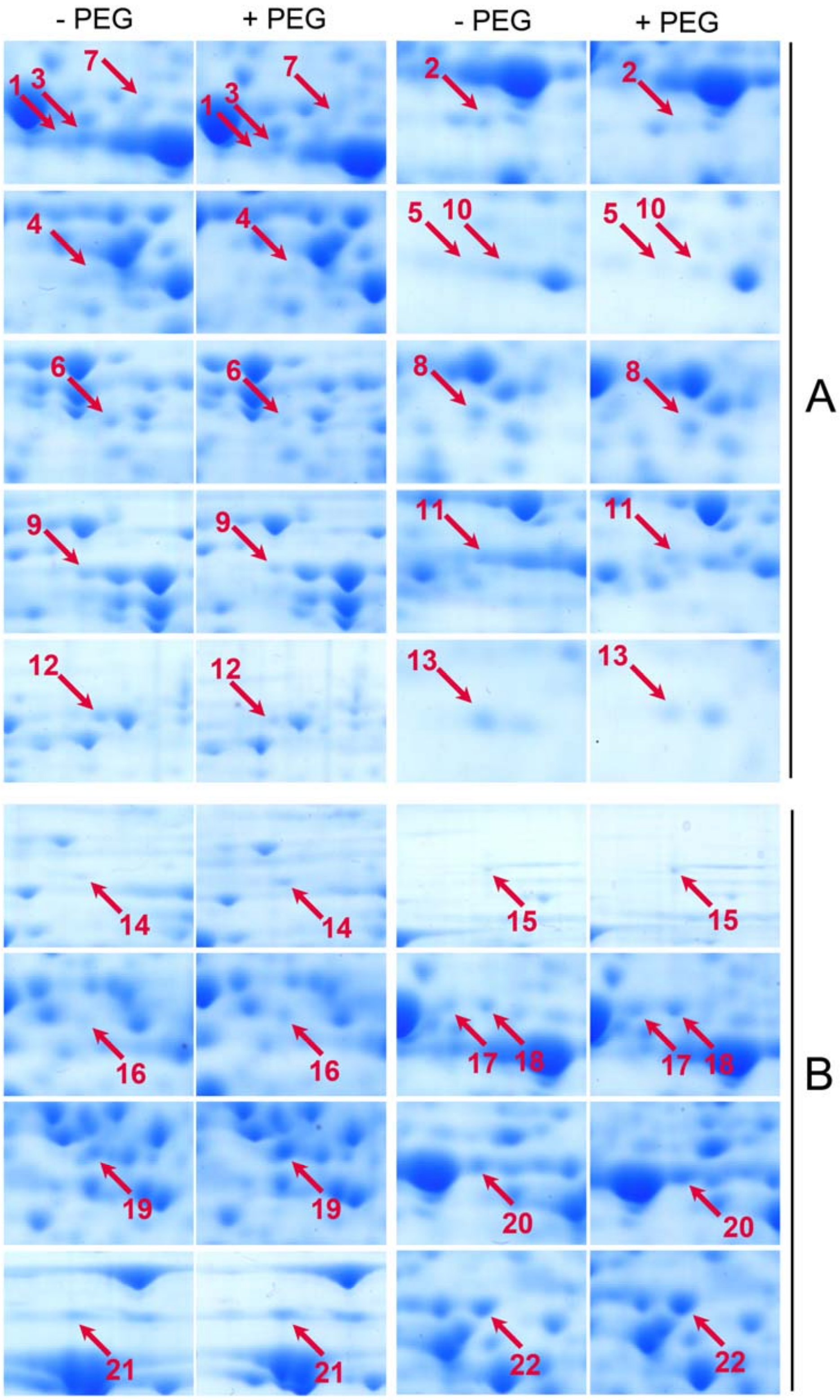

Figure S1 Close-ups of significantly decreased (A) and increased (B) protein spots in response to PEG in the root tips of common bean genotype VAX 1. The Coomassie-stained 2D IEF/SDS–PAGE gels of the total soluble proteins are shown in Fig. 2.

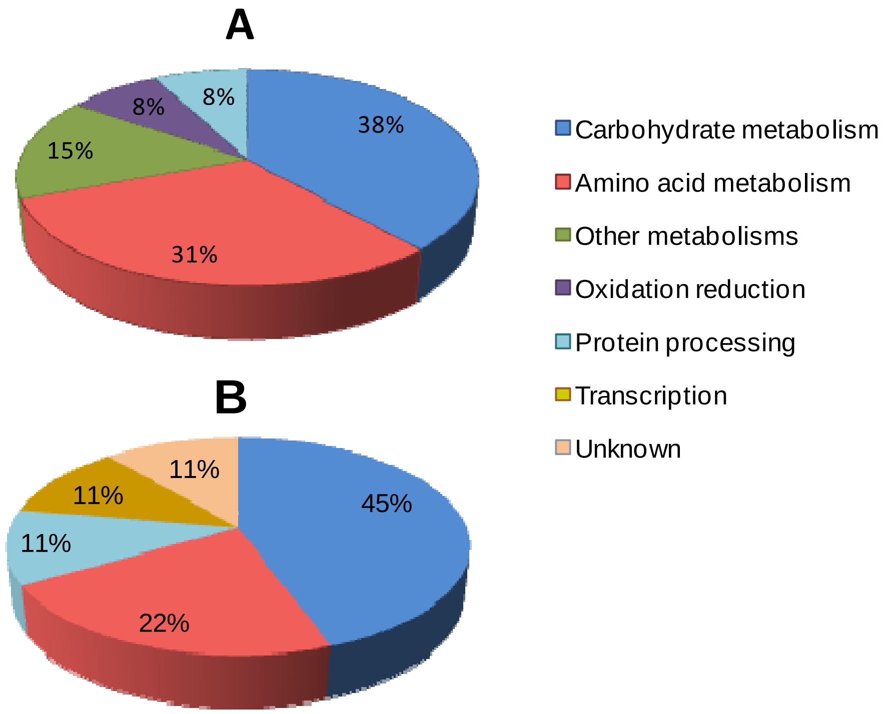

Figure S2 Functional categories of the 22 significantly decreased (A) and increased (B) proteins in response to PEG-induced osmotic stress in the root tips of common bean genotype VAX 1. The identifications of these 22 proteins by nano LC-MS/MS are shown in Table 1.

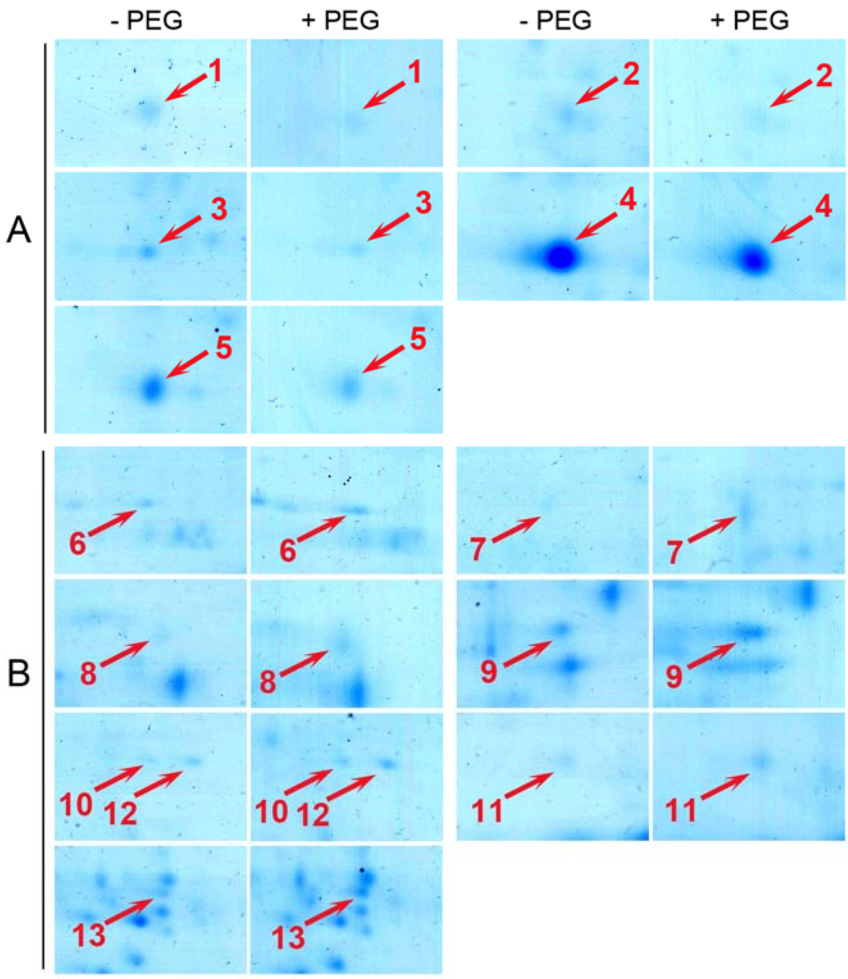

Figure S3 Close-ups of significantly decreased (A) and increased (B) apoplastic protein spots in response to PEG in the root tips of common bean genotype VAX 1. The Coomassie-stained 2D IEF/SDS–PAGE gels of the apoplastic proteins are shown in Fig. 4.

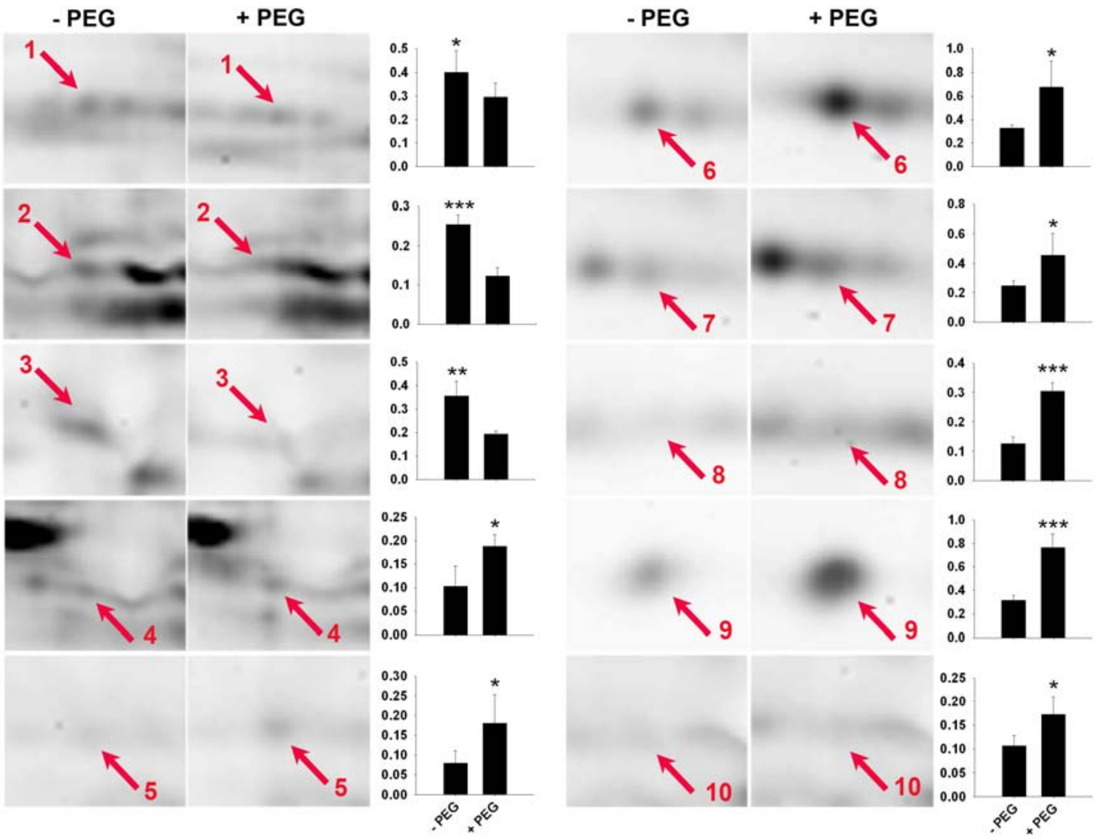

Figure S4 Close-ups and the relative volume of the 10 significantly decreased and increased phosphorylated protein spots in response to PEG in the root tips of common bean genotype VAX 1. The Pro-Q DPS-stained 2D IEF/SDS–PAGE gels of the phosphorylated proteins are shown in Fig. 5A.

```
AAB00554    MAEETQNKYE TAESSEVEVQ DRGVFDFLGK KKEEEKPQEE VIVTEFEKIT VSEEKKEEEG 60
  Spot 6    -AEETQNKYE TAESSEVEVQ DRGVFDFLGK --EEEKPQEE VIVTEFEK-- ----------
  Spot 7    -AEETQNKYE TAESSEVEVQ DRGVFDFLGK ---------- ---------- ----------
  Spot 9    -AEETQNKYE TAESSEVEVQ DR-------- ---------- ---------- ----------
AAB00554    EKKHSLLEKL HRSDSSSSSS SEEEGEDGEK KKKKKKEKKE KKKIEEKIEG YHKEDTSVPV 120
  Spot 6    ---HSLLEK- ---------- ---------- ---------- -------IEG YHKEDTSVPV
  Spot 7    ---------- ---------- ---------- ---------- ---------- ----------
  Spot 9    ---------- ---------- ---------- ---------- ---------- ----------
AAB00554    EKVEVVESEE KKGFLEKIKE KLPGHKKSEE AAAPPPPPPA ATSSEHEGEA KEKKGILEKI 180
  Spot 6    EKVEVVESEE K--------- ------KSEE AAAPPPPPPA ATSSEHEGEA K---------
  Spot 7    --VEVVESEE K--------- ------KSEE AAAPPPPPPA ATSSEHEGEA K---------
  Spot 9    ---------- ---------- ---------- ---------- ---------- ----------
AAB00554    KEKLPGYHSK TEEEKEKESG GH                                         202
  Spot 6    ---------- ---------- ---------- ---------- ---------- ----------
  Spot 7    ---------- ---------- ---------- ---------- ---------- ----------
  Spot 9    ---------- ---------- ---------- ---------- ---------- ----------
```

Figure S5 Prediction of potential phosphorylation sites of the dehydrin protein. The phosphorylation sites were analyzed with KinasePhos 2.0 software (http://kinasephos2.mbc.nctu.edu.tw/index.html, Wong et al., 2007) online. Highlighted are peptides which may undergo phosphorylation.
